# Modular 3D In Vitro Artery-Mimicking Multichannel System for Recapitulating Vascular Stenosis and Inflammation

**DOI:** 10.3390/mi12121528

**Published:** 2021-12-08

**Authors:** Minkyung Cho, Je-Kyun Park

**Affiliations:** 1 Department of Bio and Brain Engineering, Korea Advanced Institute of Science and Technology (KAIST), 291 Daehak-ro, Yuseong-gu, Daejeon 34141, Korea; mkcho25@kaist.ac.kr; 2 KAIST Institute for Health Science and Technology, 291 Daehak-ro, Yuseong-gu, Daejeon 34141, Korea

**Keywords:** 3D printing, in vitro artery-mimicking model, inflammation, modular microfluidic system, vascular stenosis

## Abstract

Inflammation and the immune response in atherosclerosis are complex processes involving local hemodynamics, the interaction of dysfunctional cells, and various pathological environments. Here, a modular multichannel system that mimics the human artery to demonstrate stenosis and inflammation and to study physical and chemical effects on biomimetic artery models is presented. Smooth muscle cells and endothelial cells were cocultured in the wrinkled surface in vivo-like circular channels to recapitulate the artery. An artery-mimicking multichannel module comprised four channels for the fabrication of coculture models and assigned various conditions for analysis to each model simultaneously. The manipulation became reproducible and stable through modularization, and each module could be replaced according to analytical purposes. A chamber module for culture was replaced with a microfluidic concentration gradient generator (CGG) module to achieve the cellular state of inflamed lesions by providing tumor necrosis factor (TNF)-α, in addition to the stenosis structure by tuning the channel geometry. Different TNF-α doses were administered in each channel by the CGG module to create functional inflammation models under various conditions. Through the tunable channel geometry and the microfluidic interfacing, this system has the potential to be used for further comprehensive research on vascular diseases such as atherosclerosis and thrombosis.

## 1. Introduction

Inflammation is a protective biological response by the immune system against harmful external stimuli such as pathogens and injuries [1]. Inadequate inflammatory processes can cause chronic inflammation that leads to diseases or tissue damage [2]. Atherosclerosis is one of the inflammatory diseases in which plaques build up to narrow an artery, and vascular inflammation is known as a key risk factor involved in atherogenesis, such as initiation, progression, leukocyte recruitment, and rupture of plaque of atherosclerosis [3,4]. When endothelial cells (ECs) are activated due to inflammation, many cytokines and chemokines are released [5]. In addition, the level of endothelial adhesion molecules is also elevated, and therefore, neutrophils, monocytes, and platelets are attached to the lesion [3,5]. Arterial stenosis and atherosclerosis can lead to various fatal diseases [6,7]; therefore, it is clinically significant to research and understand arterial problems.

A three-dimensional (3D) cell culture system with microfluidics has been improved in mimicking in vivo tissue for biological assays [8]. A 3D structure and features such as in vivo tissue recapitulating various in vivo-like microenvironments and understanding of the phenomena and mechanisms of the system can be achieved through microfluidic in vitro models [9]. Various tissues, such as blood vessels [10,11], heart [12], liver [13,14], lung [15], and intestine [16], have been mimicked and studied using microfluidics.

Many different factors, such as narrowing of blood vessels, adhesion of leukocytes and platelets, and formation of thrombi, are organically connected in atherosclerosis. In general, ECs and smooth muscle cells (SMCs) are major cell types that constitute the arterial wall [17,18]. The microchannel-based 3D models of previous studies that simulated arterial stenosis were mostly analyzed with only the stenosis structure without cells [19,20,21] or only monocultured ECs for observation of cellular phenomena, including leukocyte [22] and platelet attachment [22,23]. Since atherosclerosis is a disease that occurs in the artery, it is obviously also affected by SMCs [17]. In the in vivo-like circular channel, however, there are few studies based on the coculture of SMCs and ECs, which are two cells that mainly constitute the artery, or about stenosis. There are many studies that have performed assays with the coculture of SMCs and ECs [8], but the experiments were performed on a plane rather than in a circular channel. Moreover, studies of monocyte adhesion using SMC–EC coculture models in stenosis channels are rare. Since there is a hydrodynamic effect from the stenosis structure of the circular channel, it is necessary to simulate the structure in three dimensions rather than in a plane. Although studies of vascular inflammation and leukocyte recruitment through microfluidics have been conducted [24,25,26,27], various physical and chemical conditions have not been applied simultaneously. Menon et al. reported a perfusable 3D stenosis model to observe vascular inflammation and endothelial interaction with leukocytes [26]. However, they did not properly simulate the hydrodynamic flows in the artery using a rectangular channel. The fabricated 3D stenosis model consisted only of ECs, which cannot represent arteries without SMCs. Additionally, there was only a single channel in the device, which makes it difficult to test multiple conditions at the same time because multiple independently fabricated devices were required. In general, rectangular channels are widely used for microfluidic blood vessel models, because they can be easily fabricated by photolithography. In a rectangular channel, the shear stress at the corners is considerably low, so cells have difficulty adhering to the corners, and there are various problems such as a dead volume in a rectangular channel. Since the native blood vessel has a circular cross-section, a geometrically similar model should be fabricated. Then, the physiological conditions, such as the fluid flow acting in the channel, become similar to those of the native vessel.

**Figure 1:**
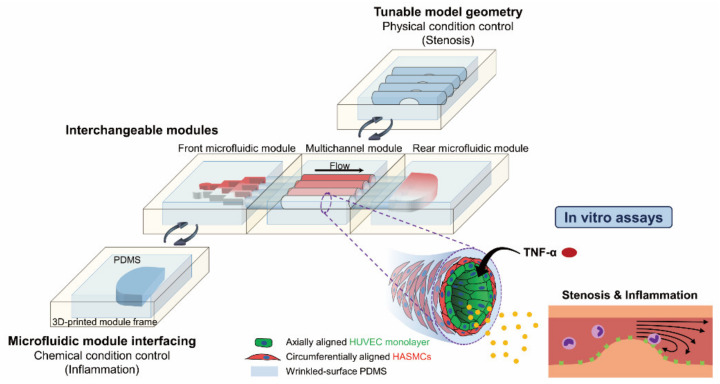
An artery-mimicking multichannel modular device for in vitro disease models. Each module, consisting of a three-dimensionally printed module frame and polydimethylsiloxane (PDMS), can be replaced, depending on the analytical purpose. The interchangeable microfluidic module interfacing enables parallel coculture model fabrication in the multichannel and chemical condition control. An in vitro artery-mimicking multichannel module with tunable model geometries for stenosis is also interchanged and used. In each channel of the in vitro artery module, axially aligned confluent human umbilical vein endothelial cell (HUVEC) monolayers and circumferentially aligned and stretched human aortic smooth muscle cells (HASMCs) are cocultured on a wrinkled surface. A modular 3D in vitro multichannel system that mimics the human artery can be used for in vitro assays to demonstrate vascular stenosis and inflammation.

In a previous study, we cocultured SMCs and ECs with in vivo-like directionality and morphology in a circular polydimethylsiloxane (PDMS) channel and studied the effect of the wall shear stress on cells with physical changes in the model by creating a stenosis structure in the channel [28]. Since hemodynamic parameters such as pressure, flow and shear stress, and cellular interactions play an important role in atherogenesis, it was necessary not only to mimic such physical conditions, but also to recapitulate the cellular state of the lesion. In this study, we demonstrated a modular 3D in vitro artery-mimicking multichannel system for recapitulating vascular stenosis and inflammation, as well as a study of the physical and chemical effects on inflammation models (Figure 1). Each module for the artery-mimicking modular device was fabricated by hybridizing the three-dimensionally printed module frame and the PDMS. Through modularization and standardization, the manipulation became reproducible and stable, and each microfluidic module could be replaced according to analytical conditions, providing the possibility of mass production. 3D human aortic smooth muscle cell–human umbilical vein endothelial cell (HASMC–HUVEC) coculture models with in vivo-like cell directionality and morphology were fabricated in parallel in the circular multichannel in one modular multichannel device with chamber modules to especially recapitulate a small muscular artery with a diameter of 1 mm. Additionally, several modular devices were simultaneously handled in a high-throughput manner. A microfluidic concentration gradient generator (CGG) module was used to recapitulate the cellular state of inflamed lesions by imparting chemical conditions to the coculture models, in addition to physical conditions such as stenosis structure and chaotic blood flow, by tuning the channel geometry. Tumor necrosis factor (TNF)-α, a cytokine that causes inflammation, was treated at multiple concentrations simultaneously in the coculture models in one artery-mimicking multichannel modular device through the CGG module to create several inflammation models. In addition, the influences of the wall shear stress, the stenosis structure, and the cell–cell interaction through the coculture of SMCs and ECs on the inflammation models were investigated to promote a comprehensive understanding of vascular diseases. The immune responses of the inflammation models were also monitored by the recruitment of monocytic THP-1 cells. That is, the developed modular multichannel system was used to create in vivo artery-like coculture models, and through tunable channel geometry and microfluidic interfacing, various conditions were assigned to the coculture models simultaneously to perform parallel biological assays for diseases.

## 2. Materials and Methods

### 2.1. Materials

Primary HASMCs, primary HUVECs, SMC growth medium-2 (SmGM-2), and endothelial cell growth medium-2 (EGM-2) were supplied from Lonza (Basel, Switzerland). THP-1 cells were purchased from the Korean Cell Line Bank (Seoul, Korea). RPMI 1640 and Dulbecco’s modified Eagle’s medium (DMEM) were ordered from Corning (New York, NY, USA), and human plasma fibronectin was purchased from Millipore (Burlington, MA, USA). Sixteen percentage formaldehyde and Hoechst 33342 were obtained from Thermo Fisher Scientific. Trichloro(1H,1H,2H,2H-perfluorooctyl)silane, erioglaucine, Triton X-100, and albumin from bovine serum (BSA) were bought from Sigma-Aldrich (St. Louis, MO, USA). Rabbit anti-smooth muscle myosin heavy chain 11 antibody (ab133567, 1:25), mouse anti-CD31 antibody conjugated with Alexa Fluor 488 (ab215911, 1:100), mouse anti-intercellular adhesion molecule 1 (ICAM1) antibody (ab2213, 1:50), rabbit anti-von Willebrand factor antibody (ab6994, 1:100), goat anti-mouse IgG H&L Alexa Fluor 488 (ab150113, 1:200), and goat anti-rabbit IgG H&L Alexa Fluor 594 (ab150080, 1:200) were ordered from Abcam (Cambridge, UK). Recombinant human TNF-α was supplied from PeproTech (Rocky Hill, NJ, USA). A PDMS polymeric base and a curing agent were purchased from Dow Chemical Company (Midland, MI, USA).

### 2.2. Fabrication of a Modular Device for In Vitro Artery Mimicking

**Figure 2:**
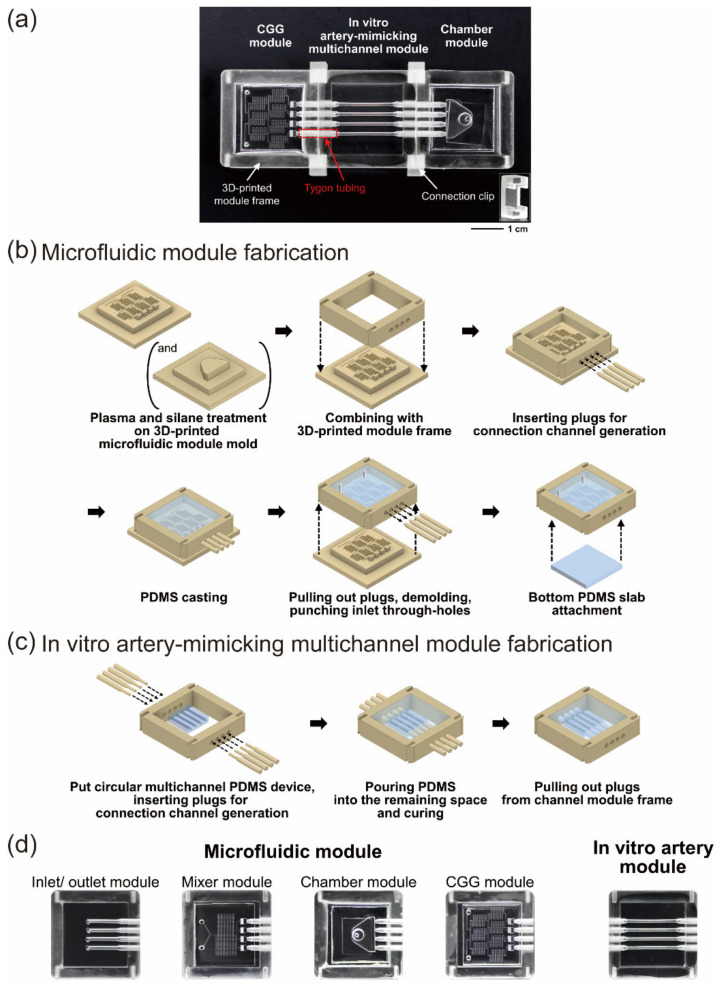
Module fabrication process of a modular three-dimensional (3D) in vitro artery-mimicking multichannel device. (**a**) Digital image of an assembled modular device. The modules were assembled using connection clips (inset). (**b**) Fabrication process of a microfluidic concentration gradient generator (CGG) module and a chamber module. (**c**) Fabrication process of an in vitro artery-mimicking multichannel module. (**d**) Fabricated microfluidic modules and an in vitro artery-mimicking module. Various microfluidic modules can be easily fabricated by changing the design of three-dimensionally printed molds, interchanged and used, depending on the analysis. Various structures of channels in the artery-mimicking multichannel module can be obtained by tuning the multichannel PDMS device design, which is put into the module in the module fabrication process.

Microfluidic modules such as the chamber module, the CGG module, and the in vitro artery-mimicking multichannel module were assembled for a modular device (Figure 2a).

Microfluidic module frames, module molds, and plugs for generating connection channels were three-dimensionally printed by PICO2 HD (Asiga, Alexandria NSW, Australia) using PlasCLEAR resin and could be used semipermanently. Both module fabrication processes are shown in Figure 2b,c. For microfluidic module fabrication, autoclaved three-dimensionally printed molds were plasma-treated for 1 min and treated with vaporized trichloro(1H,1H,2H,2H-perfluorooctyl)silane in a vacuum chamber for 1 h before casting PDMS (PDMS polymeric base and curing agent at a 10:1 mass ratio) at 65 °C for 2 h. The mold and the frame were combined, the plugs were inserted, and the PDMS was casted (Figure 2b). The diameters of the plugs used were 1.8 mm, and the part inserted into the module frame was manufactured in the shape of a truncated cone. This was because a Tygon tube with an outer diameter of 1.78 mm (AAD04127) had to be inserted between the modules, as shown in Figure 2a, and then sealed to prevent the leakage of a medium during perfusion. For the production of an in vitro artery-mimicking multichannel module, a circular multichannel PDMS device was fabricated as previously described [28]. Briefly, a PDMS device with multiple rectangle channels was casted from a three-dimensionally printed mold, and then uncured PDMS was spin-coated and cured to generate a device with multiple semicircular channels. A PDMS device with semicircular channels was stretched using a three-dimensionally printed custom-made clamp and air-plasma-treated for 30 min to produce circumferentially aligned microwrinkle structures on the channel surface. Wrinkles on the channel surface were used to promote stretching and aligning of SMCs circumferentially as in vivo through a contact guidance phenomenon. After the device release, two PDMS devices with semicircular channels were attached to fabricate a device with multiple 1-mm-diameter circular channels. The diameter of the circular channels was adjustable by a three-dimensionally printed mold channel design, and control of uncured PDMS viscosity, spin-coating speed, and time. By the 3D design of the channel mold, four-channel PDMS devices with no occlusion and 50% occlusion were fabricated for analysis. A multichannel PDMS device was placed, and plugs were inserted into the module frame to produce an artery-mimicking multichannel module (Figure 2c). The diameter of one side of the plugs used was 1.8 mm, and the diameter of the other side, which was inserted into the multichannel PDMS device, was 1.2 mm. All standardized modules of the same size were manufactured and were able to be interchanged along with the purpose of the experiment (Figure 2d).

### 2.3. HASMC–HUVEC Coculture in a Modular Device

The arterial wall consists mainly of SMCs and ECs. Primary HASMCs and HUVECs were cocultured to mimic arteries. HASMCs and HUVECs were cultured in SmGM-2 and EGM-2, respectively, in a humidified incubator with 5% CO_2_ at 37 °C. The chamber modules, the multichannel modules, the Tygon tube, and the connection clips were sterilized with 70% ethanol and then dried under ultraviolet (UV) light overnight. The in vitro artery-mimicking multichannel module was washed with phosphate-buffered saline (PBS) and coated with 50 μg/mL human plasma fibronectin at 37 °C for 1 h. After the removal of fibronectin from the channels, the multichannel module and the chamber modules were assembled using the connection clips and the Tygon tube. To prevent air from entering the channel when plugging or unplugging the module, a small amount of an extra medium was added to the module inlet, and even if a small air bubble entered, it quickly escaped from the channel through perfusion at a high flow rate. PBS and SmGM-2 medium were injected serially through a chamber module to wash multiple channels at the same time. HASMCs (1 × 10^6^ cells/mL) at passages 6 and 7 were seeded in the device and allowed to settle for 30 min. The device was flipped over, and the cells were seeded and settled down again to cover the entire channel surface. After 30 min, the device was washed by SmGM-2, and HASMCs were cultured in a humidified incubator with 5% CO_2_ at 37 °C. The medium was changed every 24 h. After 48 h, the device was washed with PBS and serum-free DMEM (1% penicillin–streptomycin). Then, 50 μg/mL fibronectin in serum-free DMEM was injected into the device and coated on the SMC layer at 37 °C for 5 min. The device was washed by PBS and EGM-2. HUVECs (4 × 10^6^ cells/mL) at passages 5 and 6 were seeded and settled down for 30 min. The seeding process was repeated with the flipped device. The channels were washed at once with a coculture medium (SmGM-2:EGM-2 volume ratio = 1:1) in the chamber module. The static coculture of cells for 24 h was followed by a 48 h perfusion coculture of cells with a wall shear stress of 0.24 Pa of a channel using a Minipuls 3 peristaltic pump (Gilson, Middleton, WI, USA). Coculture models were fabricated in parallel in multiple channels in a modular device, and four to eight modular devices were operated simultaneously. The fresh medium was changed every 24 h for static culture conditions.

The medium perfusion system was as follows. The medium flowing from a medium reservoir along the Tygon tube entered the peristaltic pump tubing (F117940, Gilson) connected by a stainless steel tube (outer diameter of 1.5 mm). Then, the medium came out through the Tygon tube connected by the stainless steel tube and flowed into the device. After perfusion, the medium went back into the reservoir.

A shear stress of 0.24 Pa, which is a low shear stress condition, was used to simulate a lesion environment where atherosclerosis occurs, such as an arterial bifurcation area and an inner curvature area with low shear stress. Since most of the analysis was performed in the straight channel here, the inflammatory condition was simulated by applying a low shear stress to the straight channels. When analyzing channels with a stenosis structure, more realistic research results can be obtained if the shear stress close to a physiological condition over 1 Pa is applied for further study. However, it could be a big challenge, because the cells may fall off the channel wall or be damaged by harsh conditions in vitro, resulting in a breakage of the cell-to-cell connection. In order to solve this problem, new coating techniques and cell culture conditions and the introduction of additional extracellular matrices (ECMs) have to be considered. Although the shear stress used in the experiment was somewhat lower than the actual range, it showed the possibility of analyzing the effect of mechanical stimuli, such as shear stress, on the artery-mimicking coculture model.

### 2.4. Characterization of the Microfluidic CGG Module

A microfluidic CGG module with a 300 μm channel width and a 100 μm channel height was fabricated. Micropillar structures (200 μm × 200 μm) were included for the efficient and complete mixing of two inlet fluids. There were four outlets for the connection with four channels of the multichannel module. It has the potential of more channels in an in vitro artery-mimicking multichannel module and a CGG module with changing three-dimensionally printed mold design. To analyze the concentration distribution ability of the CGG module, 0 and 0.6 mM erioglaucine were injected into each inlet with a flow rate of 795 μL/min per inlet, which applied 0.06 Pa to each cocultured channel. Erioglaucine solutions (0, 0.2, 0.4, and 0.6 mM) were produced by the manual pipetting method for comparison. Erioglaucine is a coloring dye with a peak absorbance at 406 nm. Ten microliters of each of the samples from each outlet of the CGG module and manual pipetting were diluted with 200 μL of distilled water, and the optical density of the diluted samples was measured by a microplate reader (VersaMax; Molecular Devices, San Jose, CA, USA).

### 2.5. TNF-α Treatment for the Inflammation Model

To investigate the adhesion molecule and the von Willebrand factor (vWF) expression in the inflammation model, an artery-mimicking multichannel system was fabricated with chamber modules under various conditions and compared: (1) static coculture; (2) perfusion coculture; (3) EC monoculture; (4) SMC–EC coculture. The front module was changed to a microfluidic CGG module for the concentration gradient distribution of TNF-α to each channel of the artery-mimicking multichannel module. Various conditions could be given simultaneously. The coculture medium and TNF-α in the coculture medium (1.5 or 15 ng/mL) were perfused to each inlet of the CGG module with a flow rate of 795 μL/min per inlet for 10 min. Then, the models were stimulated with TNF-α at 37 °C for 24 h. After TNF-α treatment, ICAM-1 and vWF were immunofluorescence-stained and analyzed for each experimental condition. The intensity measurement was made near the center of the multichannel device. Because of the cell signal overlapping at channel edges, fluorescence images excluding edges were used for the analysis.

### 2.6. Monocytic THP-1 Cell Adhesion in the Inflammation Model

Monocytic THP-1 cells were cultured in RPMI 1640 supplemented with 10% fetal bovine serum and 1% penicillin–streptomycin in a humidified incubator with 5% CO_2_ at 37 °C. THP-1 nuclei were fluorescently stained with 1 μg/mL Hoechst 33342 in RPMI 1640 at 37 °C for 15 min and then prepared at a concentration of 5 × 10^5^ cells/mL in media (SmGM-2:EGM-2:RPMI 1640 volume ratio = 1:1:1). To investigate monocyte recruitment in the inflammation model, inflammation models under various conditions were fabricated and analyzed: (1) a multichannel device with no occlusion channels in static culture conditions; (2) a multichannel device with no occlusion channels in perfusion culture conditions; (3) a multichannel stenosis device with 50% occlusion channels in perfusion culture conditions. Perfusion models were cultured with 0.24 Pa of the channel front wall shear stress. After fabricating inflammation models using the CGG module, two chamber modules were assembled back and forth to the multichannel module of the cocultured inflammation models. THP-1 cells were perfused into the device and incubated under the static conditions at 37 °C for 1 h. Channel washing with PBS and the coculture medium was followed by the fluorescence imaging of the channel area. The stained THP-1 cells in each region (upstream, inlet, apex, outlet, downstream) were counted and analyzed.

### 2.7. Immunofluorescence Staining and Imaging

To confirm the SMC–EC coculture models in the multichannel module, HASMCs and HUVECs were visualized by immunofluorescence staining of smooth muscle myosin heavy chain (SM-MHC) and platelet endothelial cell adhesion molecules (PECAM-1 or CD31), respectively. ICAM-1 and vWF were immunofluorescence-stained for the analysis of the inflammation model and monocyte adhesion which is the immune response of the inflammation model. First, the models were fixed in 4% formaldehyde for 15 min, permeabilized in 0.1% Triton X-100 for 10 min and blocked with 3% BSA in PBS for 30 min at room temperature. The cells were immunofluorescence-stained with rabbit anti-SM-MHC, mouse anti-CD31 with Alexa Fluor 488, mouse anti-ICAM1 and rabbit anti-vWF primary antibodies in 1% BSA in PBS at 4 °C overnight, and goat anti-mouse and anti-rabbit IgG antibodies in 1% BSA in PBS at room temperature for 1 h. Nuclei were stained with 5 μg/mL Hoechst 33342 in PBS at room temperature for 10 min. PBS washing for 5 min was performed three times between all processes. All phase-contrast and immunofluorescence staining images were visualized using an inverted microscope (IX51, Olympus, Tokyo, Japan).

### 2.8. Statistical Analysis

All data in graphs were presented as the mean ± standard deviation, and calculations were performed using SPSS software (version 25, IBM Corp., Armonk, NY, USA). The student’s *t*-test was used for the significance test between two conditions, and the one-way ANOVA with post hoc Tukey’s test was used for multiple comparisons. *p*-values less than 0.05, 0.01, and 0.001 were considered statistically significant.

## 3. Results and Discussion

### 3.1. Fabrication of a Modular 3D In Vitro Artery-Mimicking Multichannel System

We fabricated a module-type in vitro artery-mimicking multichannel device that was a three-dimensionally printed frame–PDMS hybrid (Figure 1). Using a modular system, arterial disease models could be simulated and analyzed. The modular device had an artery-mimicking multichannel module in the center, and microfluidic modules were connected to the front and the rear of the multichannel module using a Tygon tube and connection clips (Figure 2a). HASMCs and HUVECs formed a coculture model in each channel of the multichannel device. Circumferentially aligned wrinkle structures existed on the surface of each PDMS channel [28], and SMCs and ECs with in vivo-like directionality by wrinkles and flow were cocultured to create a model. Since cell directionality plays an important role in cell functionality, a model to recapitulate in vivo cell directionality was fabricated. Each module was of the same standardized size and could be replaced according to the purpose of the experiment. Depending on the type of microfluidic module interfacing, the fabrication of a coculture model or the control of chemical conditions such as inflammation was performed. Stenosis was simulated, compared and analyzed by the tunable model geometry of the multichannel module. Using the 3D printing of channel molds, the design of several microfluidic module channels and an artery-mimicking multichannel could be easily controlled (Figure 2b–d). As shown in Figure 2c, the wrinkled circular multichannel PDMS device fabricated as in a previous study [28], which had wrinkles with a wavelength of 5.2 ± 0.33 μm through 30 min of plasma treatment, was used to manufacture the modular device.

**Figure 3:**
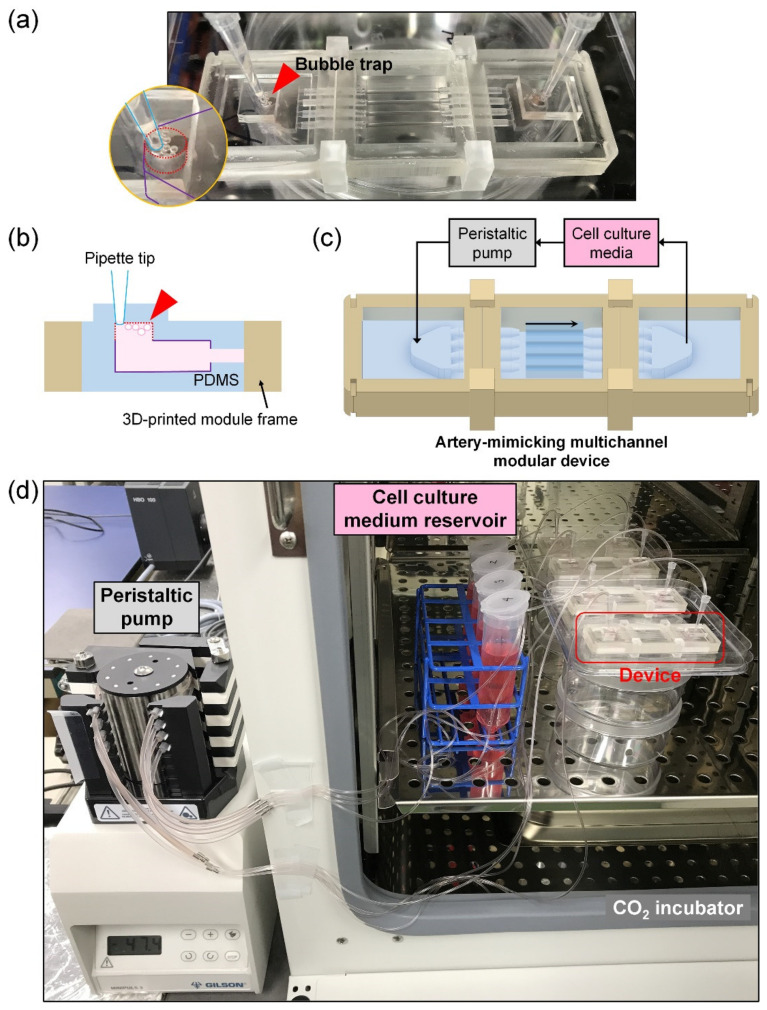
An overall operating system for modular 3D in vitro multichannel devices. (**a**) Photo of the modular 3D in vitro artery-mimicking multichannel device. The in vitro artery-mimicking multichannel module was connected with two chamber modules back and forth in a modular device for the coculture of cells. The bubble trap in the chamber module is shown in a magnified image. (**b**) Cross-sectional side view of the chamber module. The module was a hybrid of PDMS and a three-dimensionally printed frame. Bubbles, which could disturb the medium flow, block channels and interfere with the adhesion and culture of cells on the channel surface, were collected in the bubble trap. Scheme (**c**) and photograph (**d**) of the overall system. Four to eight modular devices were operated simultaneously in a high-throughput manner.

The HASMC–HUVEC coculture model was produced using a modular device assembled by connecting two chamber modules to a multichannel module (Figure 3a). There were bubble traps at the inlet and outlet parts of the chamber modules. Since bubbles can block channels, interfere with cell culture or change the proper flow patterns, bubble traps were installed in front of and rear of the channels to collect bubbles and prevent such phenomena (Figure 3b). The bubbles floated up with buoyancy, so once captured, they did not fall out. Figure 3c,d presents the establishment of a modular 3D in vitro artery-mimicking multichannel system. The in vivo hydrodynamic microenvironment, such as shear stress, was recapitulated through medium perfusion for a coculture model. Since four to eight modular devices were operated at the same time and each device had four channels, it was possible to compare and analyze multiple physical or chemical conditions, such as stenosis structure or TNF-α dose, simultaneously in a high-throughput manner. In addition, a reproducible and stable modular system for manipulation made it possible to minimize manual manipulation through modularization.

**Figure 4:**
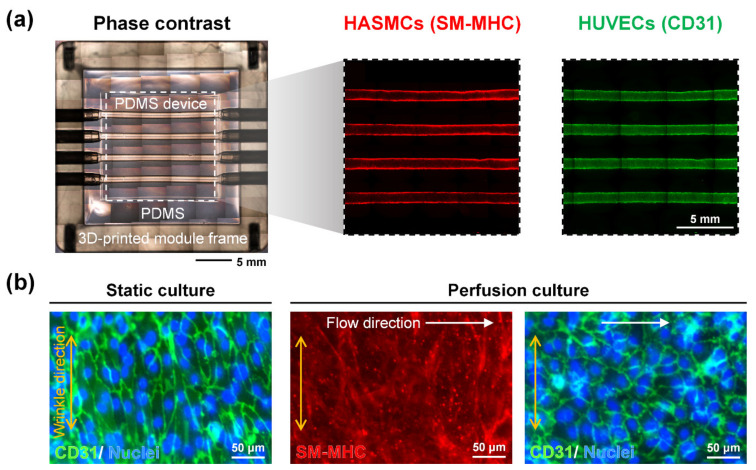
Cocultured HASMCs and HUVECs in an artery-mimicking multichannel device. (**a**) Phase-contrast image and immunofluorescence staining images of HASMCs and the HUVEC layer of the perfusion coculture model in a multichannel module. Several images were stitched for the whole device observation. (**b**) Smooth muscle myosin heavy chain (SM-MHC) and CD31 of the coculture model in static and perfusion culture conditions.

HASMCs and HUVECs were sequentially seeded and grown on the wrinkled multichannel surface of the produced modular device to form stacked cell layers to prepare an in vivo artery recapitulating coculture model (Figure 4a). Stacked two cell layers were confirmed by the cross-section image and the reconstructed 3D image of each cell layer as performed in the previous study [28]. Through the immunofluorescence staining of the SM-MHC, which is a contractile-specific marker, it was revealed that the HASMCs of the coculture model had contractility. CD31, which is an endothelial cell-specific marker, proved that there were well-formed intercellular junctions between HUVECs. Each PDMS channel had a circumferentially aligned wrinkle structure on the surface for the contact guidance of SMCs [28]. SM-MHC and CD31, markers of HASMCs and HUVECs, respectively, of the coculture model under static and perfusion culture conditions, are shown in Figure 4b. SMCs that were circumferentially aligned and stretched as in vivo and had a contractile phenotype through wrinkles were verified by SM-MHC expression. HUVECs formed a confluent monolayer on the HASMC layer. Under static conditions, HUVECs were also circumferentially aligned due to underneath circumferentially aligned HASMCs, but under perfusion conditions, a confluent layer of HUVECs axially aligned due to the medium flow was observed through CD31 staining. Since multiple channels in a device were controlled by one front microfluidic module, coculture models could be generated in parallel in each channel simultaneously, and it was then possible to analyze several conditions simultaneously in one device. Components such as stainless steel tubes were eliminated, and a more reproducible device was fabricated by modularizing the device, resulting in an augmented area for growing cells compared to that in the previous study. The wall shear stress in the device under perfusion conditions was confirmed through COMSOL simulation (Supporting Figure S1a) as in a previous report [28]. In the magnified images of a channel in the in vitro artery-mimicking multichannel module, in which HASMCs and HUVECs were cocultured, the wall shear stress and the flow velocity were maintained constant in the cell culture area (Supporting Figure S1a,b). In other words, it was confirmed that the same culture conditions were applied to each coculture model, because all the cells in the channel were subjected to uniform shear stress and flow velocity. Two types of cells with in vivo-like directionality and functionality were cocultured in a modular device to create an artery-recapitulating model.

Although native arterial vessel consists of various types of cells, ECM layers, and basement membrane, we first cocultured HASMCs and HUVECs, which mainly constitute the arterial wall, since it is quite challenging to recapitulate all of these structural components. Fibronectin was chosen for the easy coating of the channel surface for cell attachment. It was confirmed that ECMs such as laminin and collagen type IV were synthesized from cocultured cells in our previous study [28]; therefore, we believe that our model can be used to recapitulate arteries in terms of channel shapes and cell composition.

### 3.2. Concentration Gradient Generation in a Multichannel with a Microfluidic CGG Module

To comprehensively study diseases such as atherosclerosis, it is necessary to investigate not only physical conditions such as stenosis structure and shear stress, but also chemical conditions such as the cellular state and the cellular interaction of the lesion. Therefore, we tried to simulate inflammation and analyze the effects of the chemical conditions by assigning different chemical conditions to each of the artery-mimicking coculture models in the fabricated multichannel. For this purpose, a microfluidic CGG module was exploited. Micropillar structures were designed in the CGG channel to allow the complete mixing of the two inlet fluids (Supporting Figure S2a). The front microfluidic module was switched to the CGG module to give a concentration gradient to the multichannel (Supporting Figure S2b). There were two inlets in the CGG module, and two injected inlet fluids were distributed with a linear concentration gradient to each channel of the multichannel module (Supporting Figure S2c). The performance of the microfluidic CGG module was tested using erioglaucine. Erioglaucine solutions (0, 0.2, 0.4, and 0.6 mM) were prepared by the manual pipetting method and compared with the concentrations of each of the four outlet solutions of the CGG module with inlet solutions of 0 and 0.6 mM. As a result, there was no significant difference in the optical density of the resulting solutions between the manual pipetting method and the concentration gradient generation method using the CGG module (Supporting Figure S2d). In other words, the generation of a concentration gradient using the CGG module was linear between the two inlet concentrations, and further experiments were performed based on this result. Different chemical conditions were simultaneously applied to the coculture models in one device using the CGG module. In addition, external environmental conditions other than the concentration condition were maintained constant by minimizing manual manipulation, so that only the effect of the concentration condition on the models could be studied. Various types of microfluidic modules can be manufactured by new and easy design of three-dimensionally printed molds according to the experimental purpose (Figure 2d and Supporting Figure S3), which shows the potential to use artery-mimicking models for various disease analyses in the future.

### 3.3. TNF-α Dose-Dependent Adhesion Molecule and vWF Expression in an Inflammation Model

**Figure 5:**
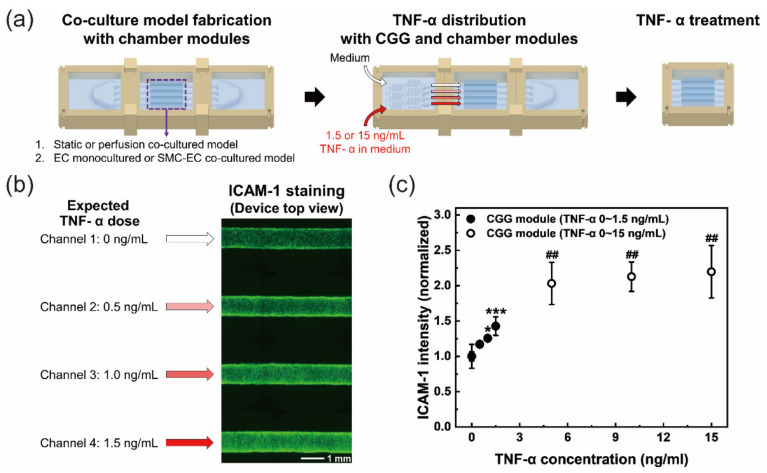
Tumor necrosis factor (TNF)-α dose-dependent inflammation model. (**a**) Experimental procedure for the inflammation model. A coculture model was fabricated with chamber modules. Static vs. perfusion coculture conditions and EC monoculture vs. SMC–EC coculture were compared. (**b**) Intercellular adhesion molecule 1 (ICAM-1) fluorescence staining images in different channels. Based on the CGG module performance, TNF-α concentrations applied to each channel in a multichannel device were estimated, and ICAM-1 expression in the models was immunofluorescence-stained and observed. (**c**) The ICAM-1 intensity of four channels in a device with one inlet TNF-α concentration of 1.5 or 15 ng/mL. Since the channel diameter was large and the whole diameter was not included in the depth of focus, we analyzed only the area of 70% of the center of the channel width, excluding the channel edge part. * *p* < 0.05, *** *p* < 0.001 vs. no treatment channel with 1.5 ng/mL inlet TNF-α; ## *p* < 0.01 vs. no treatment channel with 15 ng/mL inlet TNF-α. All *n* = 3 independent devices.

TNF-α, a proinflammatory cytokine, is involved in the innate immune response as a mediator of inflammation and the immune system [29,30]. It regulates many signaling pathways and promotes inflammation [31]. TNF-α-activated vascular models alter the expression levels of adhesion molecules and vWF [32,33]. We compared and analyzed the expression of ICAM-1 and vWF according to the TNF-α treatment concentration in the models under various culture conditions. ICAM-1, as an inflammatory adhesive cell surface protein, and vWF, as a procoagulatory protein, are associated with leukocyte recruitment and platelet aggregation in blood vessels [34]. A cellular model with in vivo-like directionality in each channel of the in vitro artery-mimicking multichannel module was fabricated using two chamber modules (Figure 5a). We constructed cellular models under various culture conditions to compare the expression of ICAM-1 and vWF: (1) static coculture model vs. perfusion coculture model, (2) EC monoculture model vs. SMC–EC coculture model. By replacing the front module from the chamber with the microfluidic CGG module, which was connected to the multichannel module with the produced cellular models, a linear TNF-α concentration gradient was formed and treated in each channel of the multichannel module. Then, the inflammatory response to TNF-α concentrations, such as ICAM-1 and vWF expression, was observed for each culture model.

When the medium and 1.5 ng/mL TNF-α in the medium were injected into the device through two inlets of the CGG module, each channel of the multichannel module was treated with estimated concentrations of TNF-α of 0, 0.5, 1, and 1.5 ng/mL based on the CGG module performance test. The higher the TNF-α concentration was, the higher the ICAM-1 expression level in the SMC–EC perfusion coculture model (Figure 5b). Depending on the concentration of the inlet fluid to be injected, TNF-α of various concentration ranges could be simultaneously treated in the multichannel. By injecting 1.5 or 15 ng/mL TNF-α into one inlet, changes in ICAM-1 expression in the models over a wide range of TNF-α doses were obtained (Figure 5c). The ICAM-1 expression level was almost saturated over a concentration of 5 ng/mL TNF-α. Because it might be difficult to clearly see the effect of TNF-α on the marker at high concentration ranges, and more sensitive results could be obtained at a low concentration range, the concentration range from 0 to 1.5 ng/mL of TNF-α was determined for use and tested further.

**Figure 6:**
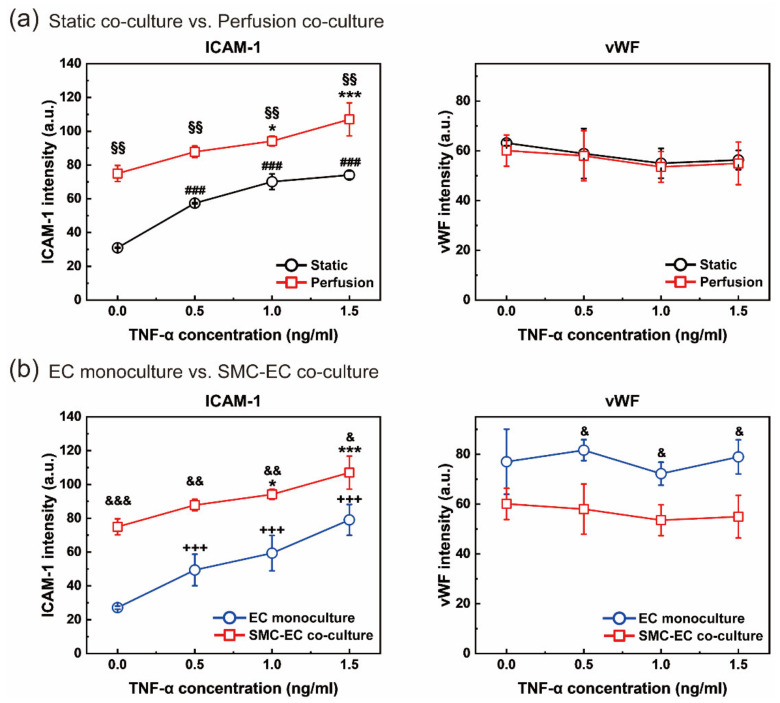
TNF-α dose-dependent ICAM-1 and von Willebrand factor (vWF) expression in the inflammation model. (**a**) Comparison of ICAM-1 and vWF expression between static and perfusion coculture conditions. For the ICAM-1 graph, §§ *p* < 0.01 vs. static culture conditions at the same TNF-α dose; * *p* < 0.05, *** *p* < 0.001 vs. no treatment channel in perfusion conditions; ### *p* < 0.001 vs. no treatment channel in static conditions. (**b**) Comparison of ICAM-1 and vWF expression between the EC monoculture and SMC–EC coculture models. & *p* < 0.05, && *p* < 0.01, &&& *p* < 0.001 vs. EC monoculture conditions at the same TNF-α dose. For the ICAM-1 graph, * *p* < 0.05, *** *p* < 0.001 vs. no treatment channel in SMC–EC coculture conditions; +++ *p* < 0.001 vs. no treatment channel in EC monoculture conditions. All *n* = 3 independent devices.

Next, cellular models fabricated under various conditions were analyzed. First, the HASMC–HUVEC coculture models in static and perfusion culture conditions were compared (Figure 6a). In general, the higher the TNF-α dose was, the higher the ICAM-1 expression level. When comparing the perfusion culture and the static culture conditions in the model fabrication process, the level of ICAM-1 in the perfusion coculture model was higher. Shear stress is known to affect ICAM-1 upregulation in ECs [35,36,37,38]. The perfusion culture model was a model in which the wall shear stress, a mechanical stimulus, was continuously applied for 48 h during culture. A low shear stress of 0.24 Pa along with TNF-α was applied to the cells, which developed a pathological environment; hence, the perfusion model expressed more ICAM-1 than the static model. We were able to observe the effects of the wall shear stress and cytokines on the SMC–EC coculture model. This result is in agreement with previous reports that studied the effects of TNF-α and shear stress on ECs [39]. In the case of vWF, there was no significant difference between static and perfusion conditions, but both conditions showed a similar tendency: the vWF expression level slightly decreased, as the TNF-α dose increased. It is known that vWF is secreted from cells and is not deposited to the ECM in a low shear stress environment, and there is less intracellular vWF in a low shear stress condition than in a static condition [33]. According to Galbusera et al. [40], when shear stress is applied to HUVECs, vWF release from ECs is significantly increased. That is, more vWF is released through exocytosis in the presence of shear stress. However, they measured the amount of vWF released to the cell supernatant and reported that the amount increased when shear stress was applied, indirectly meaning that the amount of vWF in the cell decreased. Meanwhile, we observed vWF protein through immunofluorescence staining. Since the amount of intracellular vWF was observed, the amount of stained vWF under perfusion culture conditions was slightly lower than that under static conditions, showing consistent results with a previous study. Additionally, TNF-α inhibits vWF synthesis and storage and increases vWF secretion from cells, which increases as the dose increases [33,41,42]. Therefore, due to the low shear stress and TNF-α in our experiment, both the static and perfusion culture models showed a tendency to express less vWF as the TNF-α dose increased, and the perfusion condition showed a slightly lower value. The reason for the small difference in vWF intensities between the static and perfusion culture models was that when TNF-α was not treated, the levels of vWF expression were already similar under static and perfusion conditions. According to previous reports, the higher the concentration of TNF-α, the more vWF is released, but 1.5 ng/mL is not a concentration that makes a significant difference [41]. As a result, the amount of vWF in the cells was lowered either by shear stress or by TNF-α, but the difference was insignificant within the experimental range.

Second, the EC monoculture model and the SMC–EC coculture model under perfusion conditions were compared (Figure 6b). The coculture with SMCs induced more ICAM-1 expression in ECs, regardless of TNF-α treatment. ECs cultured with proliferating secretory SMCs showed increased responsiveness to TNF-α in a previous report [43]. An atheroprone environment refers to an environment susceptible to atherosclerosis, usually at a low shear stress location such as a bifurcation. The inflammation model in this study was generated with low shear stress atheroprone flow and TNF-α treatment. Then, the proliferative SMCs caused by atheroprone conditions induced ECs to be more sensitive to TNF-α, and thus, a higher expression level of ICAM-1 in the coculture model was obtained. In the case of no TNF-α treatment, the coculture model showed higher ICAM-1 expression than the monoculture model due to the low shear stress condition. In other words, it seems that this result was probably due to the cellular interaction between SMCs and ECs. In the case of vWF, the SMC–EC coculture model became more atheroprone than the EC monoculture model due to TNF-α and the low shear stress. More vWF was released from cells and not deposited to the ECM, which was synthesized [28] in the fabricated inflammatory coculture model, so that the coculture model showed a lower intracellular vWF level.

In our experiment, the cytokine TNF-α at multiple concentrations was simultaneously applied to one multichannel device through the microfluidic CGG module, and inflammation models were constructed. The effects of the TNF-α dose, the wall shear stress, and the cell–cell interaction between SMCs and ECs on ICAM-1 and vWF expression were analyzed. As a result, parallel biological assays for diseases are possible through the microfluidic interfacing of the modular system.

### 3.4. TNF-α Dose-Dependent Monocyte Adhesion in the Inflammation Model

An activated endothelium through inflammation causes cell signaling processes such as the upregulation of adhesion molecules. Through the immune response, inflammatory immune cells such as monocytes and neutrophils are recruited and attached to the inflamed site [44,45]. Monocyte recruitment, in particular, is known to be involved in the early stage of atherogenesis [45,46,47]. To analyze monocyte adhesion to inflammation, several inflamed SMC–EC coculture models were employed: (1) a multichannel device with four channels without occlusion (0% stenosis device) in static culture conditions; (2) a multichannel device with four channels without occlusion (0% stenosis device) in perfusion culture conditions; and (3) a multichannel stenosis device with four 50% occlusion channels (50% stenosis device) in perfusion culture conditions. Based on the tunable geometry of the multichannel device and microfluidic module interfacing, it was possible to perform parallel biological assays by simultaneously assigning various physical and chemical conditions.

**Figure 7:**
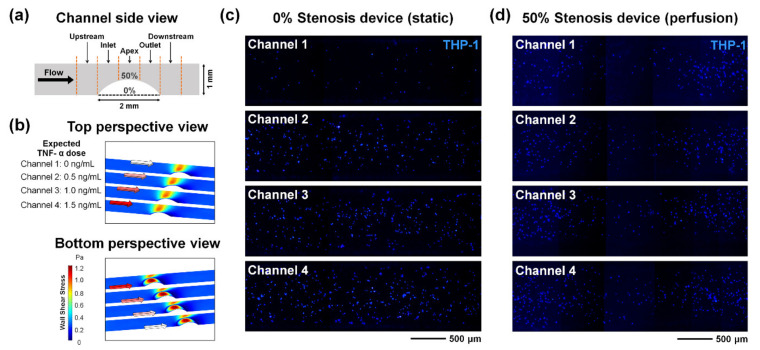
Monocytic THP-1 cell adhesion in the inflammation model. (**a**) The side view of the channel, which was divided into five regions for analysis (upstream, inlet, apex, outlet, and downstream). The presence of a flow and the presence of a stenosis structure were the parameters. (**b**) Simulated wall shear stress of a multichannel stenosis device with 50% occlusion. (**c**) The recruitment of THP-1 cells in each channel of a nonstenotic multichannel device under static culture conditions. (**d**) The recruitment of THP-1 cells in each channel of a multichannel stenosis device with 50% occlusion under perfusion culture conditions. The estimated TNF-α doses were 0, 0.5, 1, and 1.5 ng/mL from channel 1 to channel 4.

**Figure 8:**
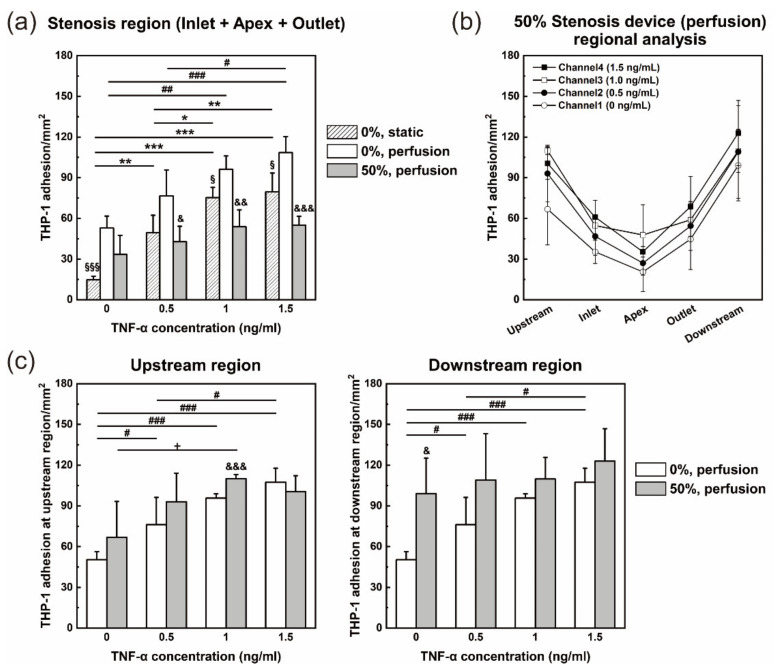
TNF-α dose-dependent monocytic THP-1 cell adhesion in the inflammation model. (**a**) THP-1 adhesion in the stenosis region of each channel of each conditioned device. * *p* < 0.05, ** *p* < 0.01, *** *p* < 0.001; # *p* < 0.05, ## *p* < 0.01, ### *p* < 0.001; § *p* < 0.05, § § § *p* < 0.001 and & *p* < 0.05, && *p* < 0.01, &&& *p* < 0.001 vs. 0% stenosis device in the perfusion condition. (**b**) Regional analysis of each stenosis channel in a 50% stenosis device in the perfusion condition. (**c**) Upstream region and downstream region analyses between a nonstenotic and a 50% stenosis device in the perfusion condition. For the upstream graph, # *p* < 0.05, ### *p* < 0.001; + *p* < 0.05; &&& *p* < 0.05. For downstream graph, # *p* < 0.05, ### *p* < 0.001; & *p* < 0.05. All *n* = 4 independent devices.

The channel area near the stenosis structure was analyzed by dividing it into five regions (upstream, inlet, apex, outlet, and downstream) (Figure 7a). The wall shear stress of a multichannel stenosis device with 50% occlusion channels in the perfusion culture condition was observed through simulation (Figure 7b). The shear stress that cells experienced was very low across the outlet and downstream regions just behind the stenosis structure. The estimated TNF-α doses from channel 1 to channel 4 were 0, 0.5, 1, and 1.5 ng/mL. Monocytic THP-1 cell recruitment in a nonstenotic multichannel device under static culture conditions and a stenotic multichannel device under perfusion culture conditions showed more THP-1 adhesion to the model with a higher TNF-α treatment concentration (Figure 7c,d). The monocyte adhesion analysis of the stenosis region (inlet + apex + outlet) of the models under three culture conditions is shown in Figure 8a. Overall, the inflammatory response occurred more strongly with increasing TNF-α dose, and the adhesion molecule ICAM-1 was more strongly expressed, so that THP-1 adhesion increased. When comparing the 0% stenosis device in the static and perfusion conditions, ICAM-1 was more strongly expressed in the coculture model with shear stress (Figure 6a), and therefore, more THP-1 cells were attached to the presheared perfusion model (Figure 8a). This is consistent with previous studies [35,48]. In other words, it might seem that the immune response, such as leukocyte adhesion, is more likely to occur when an in vivo environment, such as wall shear stress, is applied to the inflammation model. When comparing 0% and 50% stenosis devices in the perfusion conditions, fewer THP-1 cells were recruited to the 50% stenosis perfusion device. A regional analysis of a 50% stenosis device in the perfusion culture condition was performed (Figure 8b). As the TNF-α dose increased, more ICAM-1 was expressed and more THP-1 cells adhered. There was a tendency for fewer THP-1 cells to adhere to the stenosis region, particularly the apex region, than to the peripheral regions (Figure 7d and Figure 8b). This is in agreement with previous studies, indicating that ICAM-1 expression in the stenosis region was lower than that in the peripheral regions in terms of shear conditions [49,50]. For this reason, it seems that fewer THP-1 cells were attached to the stenosis region of the 50% stenosis device in the perfusion condition than in the 0% stenosis device in Figure 8a. Due to the stenosis structure, a disturbed flow occurred in the outlet and downstream regions, which developed a recirculation zone. A very low shear stress and a low velocity were formed in the recirculation zone, which triggered other signaling pathways, including the expression of adhesion molecules. As a result of the local cell response, cell damage and ICAM-1 upregulation occurred [49], and monocyte adhesion in the recirculation zone was increased (Figure 8b). That is, the local hydrodynamic flows generated by the stenosis structure impacted on the monocyte adhesion pattern.

THP-1 attachment in upstream and downstream regions of 0% and 50% stenosis devices in the perfusion condition were compared (Figure 8c). Unlike the stenosis region analysis graphs (Figure 8a), more THP-1 cells were attached in the upstream and downstream regions for the 50% stenosis perfusion device than for the 0% stenosis perfusion device. This was because the peripheral regions may have become inflammatory due to the physical stenosis structure. In addition, downstream is the recirculation region where the shear stress is very low; therefore, ICAM-1 was more strongly expressed, and THP-1 adhesion was higher than that in the upstream region. Because the cells were functional in our model, the inflammatory response and monocyte adhesion could be observed.

We conducted the experiment by spiking monocytes into the medium. There are various components in the blood, and red blood cells occupy a very large proportion of the blood. Normal red blood cells will not adhere to ICAM-1, and when plaques are ruptured, as in atherosclerosis, they will aggregate to form a thrombus. If the experiment were conducted with real blood, it would have been more difficult for monocytes to attach to the model because a large number of red blood cells would interfere. However, since monocytes attach to the vessel wall in response to inflammation, they would still show the same tendency if they were allowed to flow for a long time. Although the shear stress and the perfused liquid environment are somewhat different from the in vivo system, the tendency and response to various conditions could be seen through this experiment. We demonstrated that this system is suitable for analyzing the physical (preshearing in the coculture model fabrication and local hydrodynamic changes by stenosis structure) and chemical (SMC–EC interaction and cytokine TNF-α dose) effects on inflammation and monocyte attachment of artery-mimicking coculture models, and this system could be used for comprehensive research on atherosclerosis.

## 4. Conclusions

In this paper, a modular 3D in vitro artery-mimicking multichannel system was fabricated to study vascular stenosis and inflammation. HASMCs and HUVECs were cocultured on wrinkled-surface circular channels in an in vitro artery-mimicking multichannel module to create artery-mimicking models that recapitulated in vivo artery characteristics such as cell directionality and biological markers. Through the modularization of the device, the reproducibility and stability of the experiment were improved, and the module could be replaced according to the purpose of analysis. The microfluidic module and artery-mimicking multichannel module could be easily fabricated into various channel structures and arterial structures, respectively, by changing the design of the three-dimensionally printed mold. Several chemical conditions were simultaneously assigned to the coculture models and analyzed through the microfluidic CGG module interfacing. Changes in monocyte adhesion, as well as changes in the expression levels of ICAM-1 and vWF in the vascular inflammation model according to the TNF-α dose, were observed. Using an in vitro artery model that recapitulated the native artery through the coculture of SMCs and ECs in an in vivo-like circular channel in the analysis, not only the effects of wall shear stress, stenosis geometry, and TNF-α dose, but also the effect of the SMC–EC interaction, which was not well shown in previous studies, on vascular stenosis and inflammation were investigated in parallel. Since four channels in one device were operated simultaneously, the fabrication of coculture models or condition control was performed easily in parallel, and the models in each channel could be compared and analyzed while all external environments other than the TNF-α doses remained constant. Since inflammation and the immune response as monocytic THP-1 cell recruitment were investigated with a modular in vitro multichannel system, this system has the potential to be used for comprehensive research on vascular diseases such as atherosclerosis in the future.

## Data Availability

Not applicable.

## Supplementary Materials

The following are available online at https://www.mdpi.com/article/10.3390/mi12121528/s1, Figure S1: The wall shear stress and the velocity profile in the device under perfusion conditions through COMSOL simulation, Figure S2: Demonstration of the microfluidic concentration gradient generator (CGG) module in a device, Figure S3: 3D designs of the microfluidic module molds and digital images of the microfluidic modules.

## References

[1] Medzhitov R. (2010). Inflammation 2010: New adventures of an old flame. Cell.

[2] Chen L., Deng H., Cui H., Fang J., Zuo Z., Deng J., Li Y., Wang X., Zhao L. (2018). Inflammatory responses and inflammation-associated diseases in organs. Oncotarget.

[3] Ross R. (1999). Atherosclerosis—An inflammatory disease. N. Engl. J. Med..

[4] Libby P., Ridker P.M., Maseri A. (2002). Inflammation and atherosclerosis. Circulation.

[5] Glezeva N., Baugh J.A. (2014). Role of inflammation in the pathogenesis of heart failure with preserved ejection fraction and its potential as a therapeutic target. Heart Fail. Rev..

[6] Gimbrone M.A., García-Cardeña G. (2016). Endothelial cell dysfunction and the pathobiology of atherosclerosis. Circ. Res..

[7] Inzitari D., Eliasziw M., Gates P., Sharpe B.L., Chan R.K.T., Meldrum H.E., Barnett H.J.M. (2000). The causes and risk of stroke in patients with asymptomatic internal-carotid-artery stenosis. N. Engl. J. Med..

[8] Truskey G.A. (2010). Endothelial cell vascular smooth muscle cell co-culture assay for high throughput screening assays for discovery of anti-angiogenesis agents and other therapeutic molecules. Int. J. High Throughput Screen..

[9] Inamdar N.K., Borenstein J.T. (2011). Microfluidic cell culture models for tissue engineering. Curr. Opin. Biotechnol..

[10] Kim S., Lee H., Chung M., Jeon N.L. (2013). Engineering of functional, perfusable 3D microvascular networks on a chip. Lab Chip.

[11] Tsai M., Kita A., Leach J., Rounsevell R., Huang J.N., Moake J., Ware R.E., Fletcher D.A., Lam W.A. (2011). In vitro modeling of the microvascular occlusion and thrombosis that occur in hematologic diseases using microfluidic technology. J. Clin. Investig..

[12] Giridharan G.A., Nguyen M.-D., Estrada R., Parichehreh V., Hamid T., Ismahil M.A., Prabhu S.D., Sethu P. (2010). Microfluidic cardiac cell culture model (μCCCM). Anal. Chem..

[13] Goral V.N., Hsieh Y.-C., Petzold O.N., Clark J.S., Yuen P.K., Faris R.A. (2010). Perfusion-based microfluidic device for three-dimensional dynamic primary human hepatocyte cell culture in the absence of biological or synthetic matrices or coagulants. Lab Chip.

[14] Domansky K., Inman W., Serdy J., Dash A., Lim M.H., Griffith L.G. (2010). Perfused multiwell plate for 3D liver tissue engineering. Lab Chip.

[15] Huh D., Matthews B.D., Mammoto A., Montoya-Zavala M., Hsin H.Y., Ingber D.E. (2010). Reconstituting organ-level lung functions on a chip. Science.

[16] Sung J.H., Yu J., Luo D., Shuler M.L., March J.C. (2011). Microscale 3-D hydrogel scaffold for biomimetic gastrointestinal (GI) tract model. Lab Chip.

[17] Li M., Qian M., Kyler K., Xu J. (2018). Endothelial–vascular smooth muscle cells interactions in atherosclerosis. Front. Cardiovasc. Med..

[18] Oosterhoff L.A., Kruitwagen H.S., van Wolferen M.E., Van Balkom B.W., Mokry M., Lansu N., van den Dungen N.A., Penning L.C., Spanjersberg T.C., de Graaf J.W. (2019). Characterization of endothelial and smooth muscle cells from different canine vessels. Front. Physiol..

[19] Jung S.Y., Yeom E. (2017). Microfluidic measurement for blood flow and platelet adhesion around a stenotic channel: Effects of tile size on the detection of platelet adhesion in a correlation map. Biomicrofluidics.

[20] Ha H., Lee S.-J. (2013). Hemodynamic features and platelet aggregation in a stenosed microchannel. Microvasc. Res..

[21] Li M., Hotaling N.A., Ku D.N., Forest C.R. (2014). Microfluidic thrombosis under multiple shear rates and antiplatelet therapy doses. PLoS ONE.

[22] Menon N.V., Tay H.M., Wee S.N., Li K.H.H., Hou H.W. (2017). Micro-engineered perfusable 3D vasculatures for cardiovascular diseases. Lab Chip.

[23] Mannino R.G., Myers D.R., Ahn B., Wang Y., Rollins M., Gole H., Lin A.S., Guldberg R.E., Giddens D.P., Timmins L.H. (2015). Do-it-yourself in vitro vasculature that recapitulates in vivo geometries for investigating endothelial-blood cell interactions. Sci. Rep..

[24] Thomas A., Daniel Ou-Yang H., Lowe-Krentz L., Muzykantov V.R., Liu Y. (2016). Biomimetic channel modeling local vascular dynamics of pro-inflammatory endothelial changes. Biomicrofluidics.

[25] van Dijk C.G.M., Brandt M.M., Poulis N., Anten J., van der Moolen M., Kramer L., Homburg E.F.G.A., Louzao-Martinez L., Pei J., Krebber M.M. (2020). A new microfluidic model that allows monitoring of complex vascular structures and cell interactions in a 3D biological matrix. Lab Chip.

[26] Menon N.V., Tay H.M., Pang K.T., Dalan R., Wong S.C., Wang X., Li K.H.H., Hou H.W. (2018). A tunable microfluidic 3D stenosis model to study leukocyte-endothelial interactions in atherosclerosis. APL Bioeng..

[27] Menon N.V., Su C., Pang K.T., Phua Z.J., Tay H.M., Dalan R., Wang X., Li K.H.H., Hou H.W. (2020). Recapitulating atherogenic flow disturbances and vascular inflammation in a perfusable 3D stenosis model. Biofabrication.

[28] Cho M., Park J.-K. (2020). Fabrication of a Perfusable 3D In Vitro Artery-Mimicking Multichannel System for Artery Disease Models. ACS Biomater. Sci. Eng..

[29] Urschel K., Cicha I. (2015). TNF-α in the cardiovascular system: From physiology to therapy. Int. J. Interferon Cytokine Mediat. Res..

[30] Ott L.W., Resing K.A., Sizemore A.W., Heyen J.W., Cocklin R.R., Pedrick N.M., Woods H.C., Chen J.Y., Goebl M.G., Witzmann F.A. (2007). Tumor necrosis factor-α-and interleukin-1-induced cellular responses: Coupling proteomic and genomic information. J. Proteome Res..

[31] Holbrook J., Lara-Reyna S., Jarosz-Griffiths H., McDermott M.F. (2019). Tumour necrosis factor signalling in health and disease. F1000Research.

[32] Sawa Y., Sugimoto Y., Ueki T., Ishikawa H., Sato A., Nagato T., Yoshida S. (2007). Effects of TNF-α on leukocyte adhesion molecule expressions in cultured human lymphatic endothelium. J. Histochem. Cytochem..

[33] Sun R.J., Muller S., Wang X., Zhuang F.Y., Stoltz J.F. (2000). Regulation of von willebrand factor of human endothelial cells exposed to laminar flows: An in vitro study. Clin. Hemorheol. Microcirc..

[34] Chiu J.-J., Chien S. (2011). Effects of disturbed flow on vascular endothelium: Pathophysiological basis and clinical perspectives. Physiol. Rev..

[35] Chiu J.J., Wung B.S., Shyy J.Y.J., Hsieh H.J., Wang D.L. (1997). Reactive oxygen species are involved in shear stress-induced intercellular adhesion molecule-1 expression in endothelial cells. Arterioscler. Thromb. Vasc. Biol..

[36] Jilkova Z.M., Lisowska J., Manet S., Verdier C., Deplano V., Geindreau C., Faurobert E., Albigès-Rizo C., Duperray A. (2014). CCM proteins control endothelial β1 integrin dependent response to shear stress. Biol. Open.

[37] Tsuboi H., Ando J., Korenaga R., Takada Y., Kamiya A. (1995). Flow stimulates ICAM-1 expression time and shear stress dependently in cultured human endothelial cells. Biochem. Biophys. Res. Commun..

[38] Nagel T., Resnick N., Atkinson W.J., Dewey C.F., Gimbrone M.A. (1994). Shear stress selectively upregulates intercellular adhesion molecule-1 expression in cultured human vascular endothelial cells. J. Clin. Investig..

[39] Chiu J.-J., Lee P.-L., Chen C.-N., Lee C.-I., Chang S.-F., Chen L.-J., Lien S.-C., Ko Y.-C., Usami S., Chien S. (2004). Shear stress increases ICAM-1 and decreases VCAM-1 and E-selectin expressions induced by tumor necrosis factor-α in endothelial cells. Arterioscler. Thromb. Vasc. Biol..

[40] Galbusera M., Zoja C., Donadelli R., Paris S., Morigi M., Benigni A., Figliuzzi M., Remuzzi G., Remuzzi A. (1997). Fluid shear stress modulates von Willebrand factor release from human vascular endothelium. Blood.

[41] Matsushita K., Morrell C.N., Lowenstein C.J. (2004). Sphingosine 1-phosphate activates Weibel-Palade body exocytosis. Proc. Natl. Acad. Sci. USA.

[42] Li Y., Li L., Dong F., Guo L., Hou Y., Hu H., Yan S., Zhou X., Liao L., Allen T.D. (2015). Plasma von Willebrand factor level is transiently elevated in a rat model of acute myocardial infarction. Exp. Ther. Med..

[43] Rainger G.E., Nash G.B. (2001). Cellular pathology of atherosclerosis: Smooth muscle cells prime cocultured endothelial cells for enhanced leukocyte adhesion. Circ. Res..

[44] Chen Z., Tang M., Huang D., Jiang W., Li M., Ji H., Park J., Xu B., Atchison L.J., Truskey G.A. (2018). Real-time observation of leukocyte–endothelium interactions in tissue-engineered blood vessel. Lab Chip.

[45] Čejková S., Králová-Lesná I., Poledne R. (2016). Monocyte adhesion to the endothelium is an initial stage of atherosclerosis development. Cor Vasa.

[46] Mestas J., Ley K. (2008). Monocyte-endothelial cell interactions in the development of atherosclerosis. Trends Cardiovasc. Med..

[47] Herbin O., Regelmann A.G., Ramkhelawon B., Weinstein E.G., Moore K.J., Alexandropoulos K. (2016). Monocyte adhesion and plaque recruitment during atherosclerosis development is regulated by the adapter protein Chat-H/SHEP1. Arterioscler. Thromb. Vasc. Biol..

[48] Srigunapalan S., Lam C., Wheeler A.R., Simmons C.A. (2011). A microfluidic membrane device to mimic critical components of the vascular microenvironment. Biomicrofluidics.

[49] Yin W., Shanmugavelayudam S.K., Rubenstein D.A. (2011). The effect of physiologically relevant dynamic shear stress on platelet and endothelial cell activation. Thromb. Res..

[50] Meza D., Musmacker B., Steadman E., Stransky T., Rubenstein D.A., Yin W. (2019). Endothelial cell biomechanical responses are dependent on both fluid shear stress and tensile strain. Cell. Mol. Bioeng..

