# Supplementary material for: Modular 3D In Vitro Artery-Mimicking Multichannel System for Recapitulating Vascular Stenosis and Inflammation
Source: Micromachines (Basel). 2021 Dec 8;12(12):1528. doi: 10.3390/mi12121528 (PMC8709401; doi:10.3390/mi12121528)
Supplement: Supplementary file 1 [file micromachines-12-01528-s001.zip › micromachines-1484929-supplementary.pdf]

Supplementary Materials

Modular 3D In Vitro Artery-Mimicking Multichannel System for Recapitulating Vascular Stenosis and Inflammation

Minkyung Cho[1] and Je-Kyun Park[1,2,*]

[1] Department of Bio and Brain Engineering, Korea Advanced Institute of Science and Technology (KAIST), 291 Daehak-ro, Yuseong-gu, Daejeon 34141, Republic of Korea
[2] KAIST Institute for Health Science and Technology, 291 Daehak-ro, Yuseong-gu, Daejeon 34141, Republic of Korea
[*] Correspondence: Correspondence: Je-Kyun Park (jekyun@kaist.ac.kr)

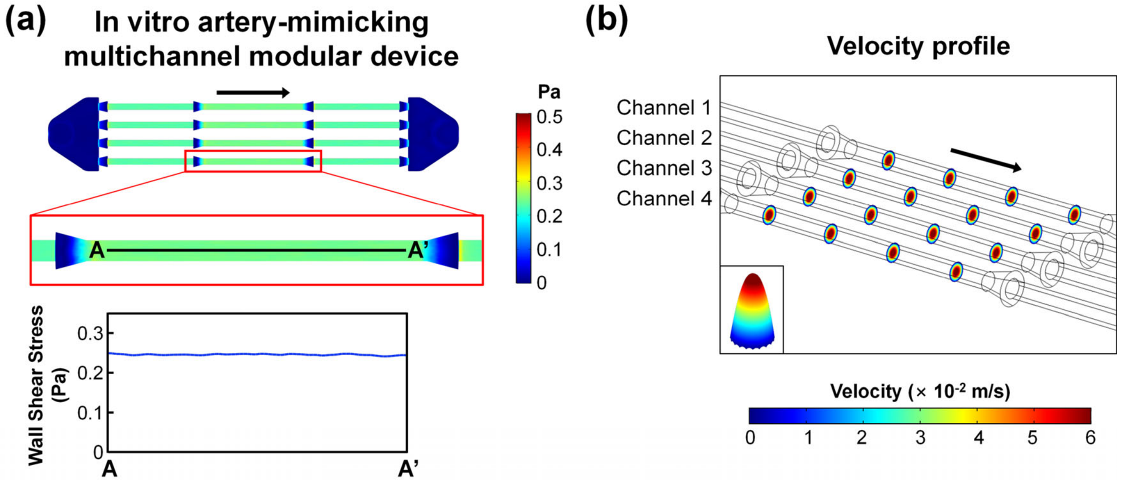

Figure S1. The wall shear stress and the velocity profile in the device under perfusion conditions through COMSOL simulation. (**a**) The wall shear stress of the device with perfusion. The channel area where cells were cocultured (A–A′) was magnified. In the culture area, the wall shear stress was maintained constant, and the cells in the channel experienced the uniform condition. (**b**) Simulation results of the cross-sectional velocity profiles in the channels and the surface velocity profile of that cross-section (inset). Briefly, COMSOL Multiphysics (Ver. 5.5, COMSOL Inc.) was used to simulate the wall shear stress of an in vitro artery-mimicking multichannel modular device and a multichannel stenosis device with 50% occlusion channels. The entire channels and device geometries including the microfluidic chamber modules were set in full three dimensions. The medium was assumed to be incompressible water, and a fine element mesh was constructed. The laminar flow physics, the inlet boundary condition of the normal inflow velocity, and the outlet boundary condition of the constant atmospheric pressure were applied. The wall shear stress was calculated and presented as a colored distribution.

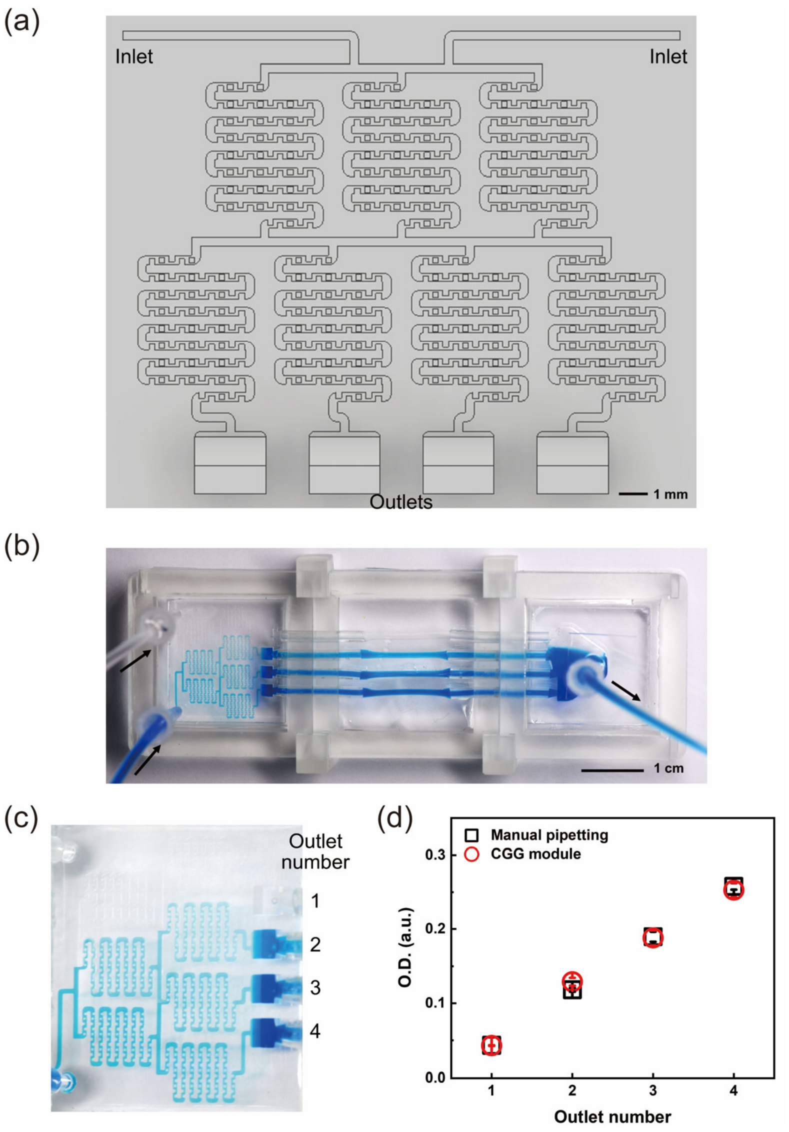

Figure S2. Demonstration of the microfluidic concentration gradient generator (CGG) module in a device. (**a**) Design of the microfluidic CGG channel. Micropillar structures were presented in the CGG channel for thorough the mixing of the fluids. (**b**) An assembled modular device with a microfluidic CGG module. The linear concentration gradient was generated by the CGG module and distributed to the multichannel in an in vitro artery-mimicking multichannel module. (**c**) A magnified image of concentration gradient generation from two inlet fluids to four outlet concentrations. (**d**) Comparison of the optical density of the generated solutions from the CGG module and the manual pipetting method. n = 3 independent devices.

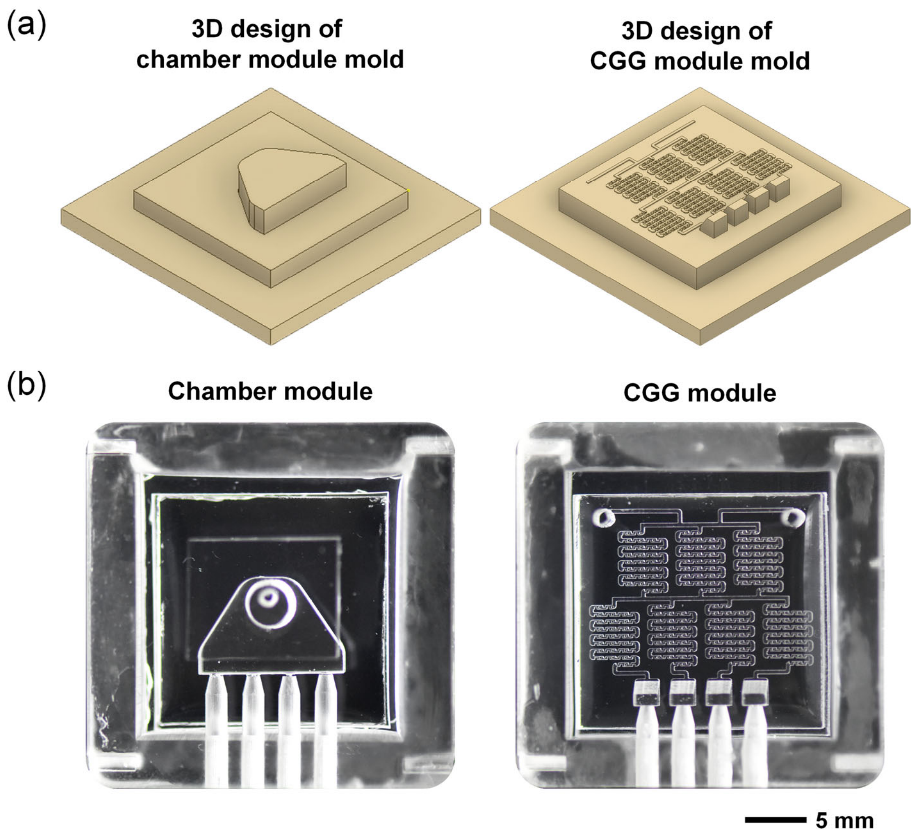

Figure S3. 3D designs of the microfluidic module molds and digital images of the microfluidic modules. (**a**) Magnified perspective view images of the 3D design of the chamber module mold and the CGG module mold. (**b**) Magnified digital images of the fabricated chamber module and the CGG module. .
